# *Acinetobacter baumannii* Genes Required for Bacterial Survival during Bloodstream Infection

**DOI:** 10.1128/mSphere.00013-15

**Published:** 2015-11-04

**Authors:** Sargurunathan Subashchandrabose, Sara Smith, Valerie DeOrnellas, Sebastien Crepin, Monica Kole, Carina Zahdeh, Harry L. T. Mobley

**Affiliations:** Department of Microbiology and Immunology, University of Michigan Medical School, Ann Arbor, Michigan, USA; Swiss Federal Institute of Technology Lausanne (EPFL)

**Keywords:** *Acinetobacter baumannii*, ATCC17978, bacteremia, fitness genes, TraDIS

## Abstract

*Acinetobacter baumannii* is emerging as a leading global multiple-antibiotic-resistant nosocomial pathogen. The identity of genes essential for pathogenesis in a mammalian host remains largely unknown. Using transposon-directed insertion-site sequencing (TraDIS), we identified *A. baumannii* genes involved in bacterial survival in a leukopenic mouse model of bloodstream infection. Mice were inoculated with a pooled transposon mutant library derived from 109,000 mutants, and TraDIS was used to map transposon insertion sites in the genomes of bacteria in the inoculum and of bacteria recovered from mouse spleens. Unique transposon insertion sites were mapped and used to calculate a fitness factor for every insertion site based on its relative abundance in the inoculum and postinfection libraries. Eighty-nine transposon insertion mutants that were underrepresented after experimental infection in mice compared to their presence in the inocula were delineated as candidates for further evaluation. Genetically defined mutants lacking *feoB* (ferrous iron import), *ddc* (d-ala-d-ala-carboxypeptidase), and *pntB* (pyridine nucleotide transhydrogenase subunit) exhibited a fitness defect during systemic infection resulting from bacteremia. *In vitro*, these mutants, as well as a *fepA* (ferric enterobactin receptor) mutant, are defective in survival in human serum and within macrophages and are hypersensitive to killing by antimicrobial peptides compared to the survival of the parental strain under these conditions. Our data demonstrate that FepA is involved in the uptake of exogenous enterobactin in *A. baumannii*. Genetic complementation rescues the phenotypes of mutants in assays that emulate conditions encountered during infection. In summary, we have determined novel *A. baumannii* fitness genes involved in the pathogenesis of mammalian infection.

**IMPORTANCE** *A. baumannii* is a significant cause of bacterial bloodstream infection in humans. Since multiple antibiotic resistance is becoming more common among strains of *A. baumannii*, there is an urgent need to develop novel tools to treat infections caused by this dangerous pathogen*.* To develop knowledge-guided treatment approaches for *A. baumannii*, a thorough understanding of the mechanism by which this pathogen causes bloodstream infection is required. Here, using a mouse model of infection, we report the identification of *A. baumannii* genes that are critical for the ability of this pathogen to cause bloodstream infections. This study lays the foundation for future research on *A. baumannii* genes that can be targeted to develop novel therapeutics against this emerging human pathogen.

## Abstract (precis)

*A. baumannii* is a significant cause of bacterial bloodstream infection in humans. Since multiple antibiotic resistance is becoming more common among strains of *A. baumannii*, there is an urgent need to develop novel tools to treat infections caused by this dangerous pathogen*.* To develop knowledge-guided treatment approaches for *A. baumannii*, a thorough understanding of the mechanism by which this pathogen causes bloodstream infection is required. Here, using a mouse model of infection, we report the identification of *A. baumannii* genes that are critical for the ability of this pathogen to cause bloodstream infections. This study lays the foundation for future research on *A. baumannii* genes that can be targeted to develop novel therapeutics against this emerging human pathogen.

## INTRODUCTION

The Gram-negative bacterium *Acinetobacter baumannii*, formerly considered an opportunistic pathogen of weak virulence, has established itself as an important hospital pathogen over the last three decades (1–3). In hospitals and convalescence units, patients typically suffer from infections of the respiratory tract, bloodstream, wounds, urinary tract, and peritoneum (4). Most alarming is the rapid rise in multiple-antibiotic-resistant *A. baumannii* strains, which limits the antibiotics available for treatment (5–7). Despite the availability of complete genome sequences for multiple strains of *A. baumannii*, the identity of most genes involved in fitness during infection of a mammalian host is not known. A comprehensive understanding of the mechanisms by which *A. baumannii* colonizes the host, avoids the immune response, and incites tissue damage within the host is critical to develop the next generation of therapeutics against this important pathogen.

High-throughput sequencing has enabled mapping of transposon (Tn) insertion sites at a population level and has been applied to detect bacterial genes involved in pathogenesis (8). Previously, we developed a murine model of experimental bloodstream infection with extraintestinal pathogenic *Escherichia coli* (9) and utilized this model to identify fitness factors involved in the survival and growth of extraintestinal pathogenic *E. coli* during systemic infection (10). Using a random transposon mutant library of *A. baumannii*, a mouse model of *A. baumannii* bloodstream infection, and high-throughput DNA sequencing-enabled transposon insertion site mapping, we report the identification of novel fitness determinants that enable *A. baumannii* to colonize and disseminate within a mammalian host. Furthermore, independent verification of fitness genes was conducted in the murine model using defined *A. baumannii* mutant strains and complementary *in vitro* assays. In summary, we present the results of a genome-wide forward genetic screen that has illuminated *A. baumannii* fitness genes during bloodstream infection and subsequent systemic colonization.

## RESULTS

### A murine model of *A. baumannii* bacteremia.

**FIG 1:**
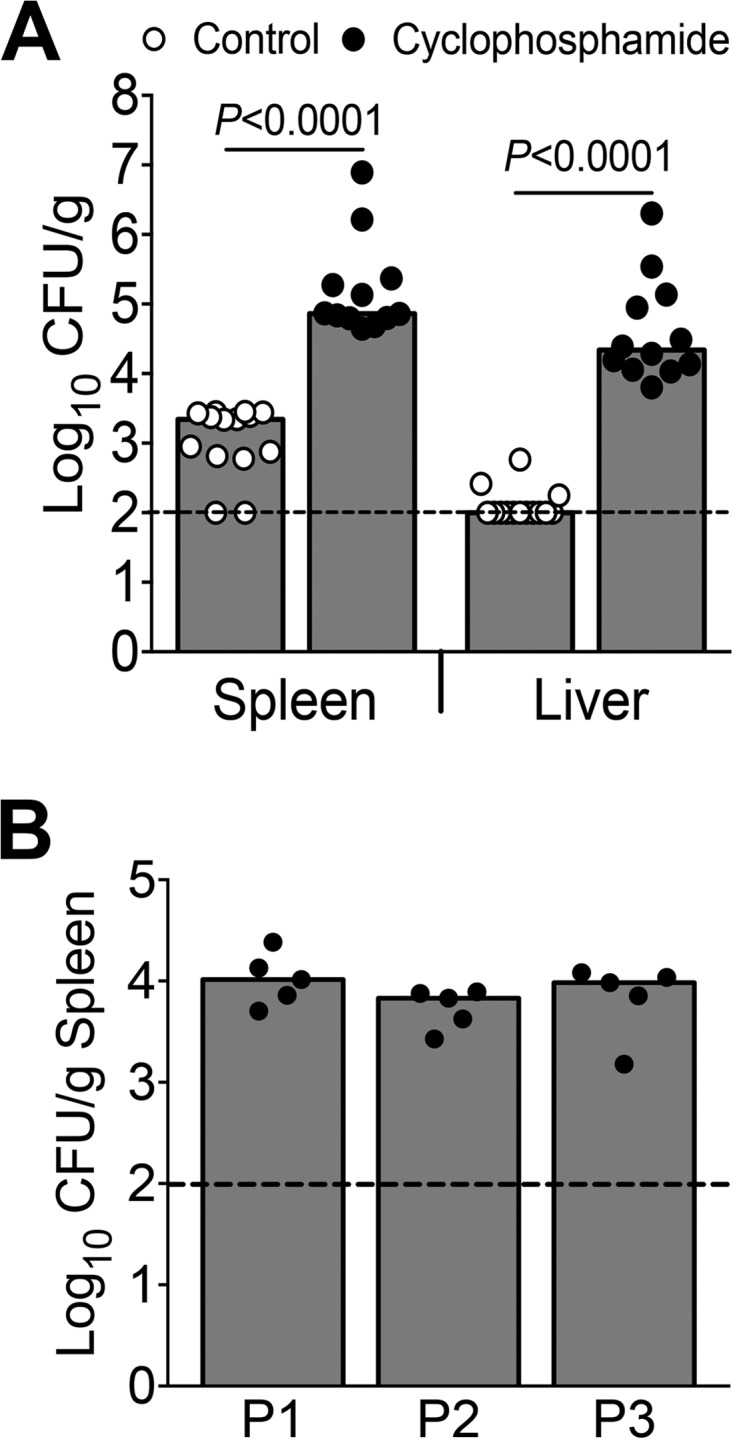
Leukopenic mouse model of *A. baumannii* infection. (A) CBA/J mice pretreated with cyclophosphamide (CP) to induce leukopenia (*n* = 12) and controls (*n* = 14) were inoculated with 10^7^ CFU of *A. baumanii* strain ATCC 17978 via tail vein. Bacterial loads in spleen and liver at 24 hpi are depicted. *P* values were determined using a two-tailed Mann-Whitney test. (B) CP-treated mice (*n* = 5/passage) were inoculated with 10^7^ CFU of *A. baumanii* strain ATCC 17978 Tn*5* mutants derived from a pool of 109,000 independent transformants. Colonies derived from spleens were pooled and used as the inoculum for subsequent passages. P1, P2, and P3 indicate the three passages. Each circle represents the result for one mouse, bars correspond to the median values, and dotted lines indicate the limit of detection.

Bloodstream infection is a common clinical manifestation of *A. baumannii* infection in humans (3). However, relatively little is known about *A. baumannii* fitness factors involved in bloodstream infection. A leukopenic mouse model emulates the immune status of a subset of immunocompromised hosts, who are at high risk for *A. baumannii* infection. Panleukopenia was experimentally induced in mice using cyclophosphamide (CP) (11). Differential blood counts revealed that CP-treated mice had 79% lower total leukocytes, 63% lower neutrophils, 80% lower lymphocytes, and 87% lower monocytes at 2 days after treatment than prior to administering CP. CP-treated and control mice were inoculated with 1 × 10^7^ CFU wild-type *A. baumannii* strain ATCC 17978 via tail vein, and tissue bacterial loads in the spleen and liver were determined at 24 h postinoculation (hpi). The spleens and livers of leukopenic mice were consistently colonized at higher numbers than those of the untreated control group, indicating that CP-induced leukopenia exacerbates systemic infection with *A. baumannii* (Fig. 1A).

### Screening of *A. baumannii* transposon mutants in a murine model.

Leukopenic mice were inoculated intravenously with 10^7^ CFU of *A. baumannii* Tn*5* transformants via the tail vein. Mice were euthanized at 24 hpi, and bacteria surviving in the spleen were enumerated. The splenic bacterial load in mice colonized with the transposon mutant pool was comparable to that in mice colonized with the wild-type strain (Fig. 1B). Entire spleens from all mice were processed for culture, colonies derived from all five spleens were pooled and used as the inoculum for a second round of infection, and the output from the second infection experiment (*n* = 5) was used as the inoculum for the third set of infection experiments (*n* = 5). In total, the transposon mutant pools were passaged through mice three times (Fig. 1B).

### Identification of putative *A. baumannii* fitness factors involved in bacteremia.

**FIG 2:**
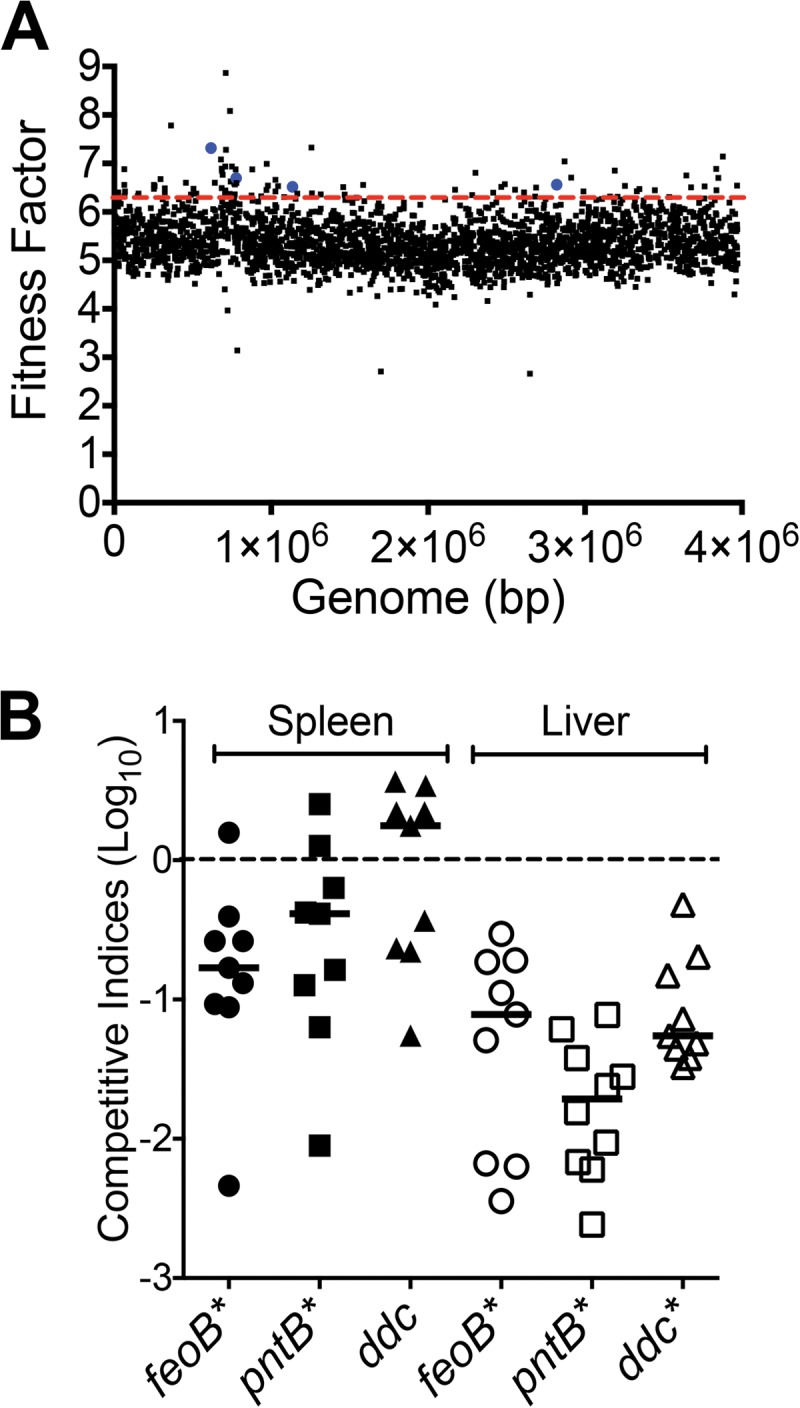
Identification and validation of bacteremia fitness genes. (A) Genome-wide distribution of fitness factors for all transposon insertion sites determined in the primary screen is depicted. Dotted red line indicates the mean + 2 standard deviations, which was used as the threshold to delineate candidate fitness genes. Blue dots indicate fitness factors for transposon mutants that were selected for further investigation. (B) Leukopenic mice were infected with a 1:1 mixture of wild-type and *feoB*, *pntB*, or *ddc* mutant strains via tail vein. Competitive indices were determined from bacterial CFU counts in spleen and liver. Bars indicate the median competitive index for each organ, and each symbol represents the competitive index for one mouse. *P* values were determined by Wilcoxon signed-rank test (*, <0.01).

In total, 2,614 unique Tn insertion sites were mapped to the genome, and the mean fitness factor was 5.4 (see Table S1 in the supplemental material). As expected for Tn*5* transposons, insertion sites were uniformly distributed across the genome, indicating the lack of hot spots (Fig. 2A). Fitness factor values >1 indicate that mutants exhibited reduced fitness during growth within the host. A conservative threshold of ≥6.3 (mean + 2 standard deviations) was used to identify 89 candidate Tn mutants that exhibit a fitness defect during bacteremia and subsequent colonization of the spleen in a mouse model (Fig. 2A; see also Table S2).

10.1128/mSphere.00013-15.1 Table S1  Fitness factors for all transposon insertion sites. Download Table S1, XLSX file, 0.1 MB. Copyright © 2015 Subashchandrabose et al. 2015 Subashchandrabose et al. This content is distributed under the terms of the Creative Commons Attribution 4.0 International license.

10.1128/mSphere.00013-15.2 Table S2  Candidate bacteremia fitness genes. Download Table S2, XLSX file, 0.04 MB. Copyright © 2015 Subashchandrabose et al. 2015 Subashchandrabose et al. This content is distributed under the terms of the Creative Commons Attribution 4.0 International license.

### *A. baumannii* candidate fitness genes involved in bacteremia.

At least one Tn*5* insertion site was identified in 59 genes, including 14 genes encoding hypothetical proteins, and in 27 intergenic regions that could affect the expression of downstream genes (see Table S2 in the supplemental material). Genes involved in the biosynthesis and maintenance of lipopolysaccharide (*A1S_0055*) and peptidoglycan (*A1S_2768*, *A1S_3334*, *A1S_3335*, and *A1S_1987*) were identified in the primary screen. During bacteremia, import of ferrous iron (*feoB-A1S_0653*) and ferric enterobactin (*fepA-A1S_0980*) appears to contribute to the fitness of *A. baumannii*. Additionally, several genes involved in transport across the membrane, transcriptional regulation, two-component signal transduction systems, and metabolism or encoding hypothetical proteins were identified as candidate fitness genes in the primary screen (see Table S2). Since a population-based primary screen was used to identify fitness factors, independent verification using genetically defined mutants lacking candidate fitness genes was performed to validate and extend the findings of the primary screen.

### Validation of primary screen using defined mutants and coinfection.

Genes that were previously not reported to be involved in the pathogenesis of *A. baumannii* infection and that encode surface components with potential for direct involvement in host-pathogen interaction were selected for further investigation. Defined, kanamycin-resistant mutants were constructed in *A. baumannii* strain ATCC 17978 using homologous recombination (12) and recombineering approaches (13). Deletion-disruption mutations were introduced in the following genes: *feoB*, encoding a ferrous iron transporter subunit (*A1S_0653*); *pntB*, encoding a pyridine nucleotide transhydrogenase subunit (*A1S_0568*); and *ddc*, encoding a d-alanine-d-alanine carboxypeptidase (*A1S_2435*). During culture in rich medium, *feoB*, *pntB*, and *ddc* mutants exhibited growth kinetics that were indistinguishable from that of the parental strain, indicating that these genes are not required for optimal growth *in vitro* (see Fig. S1 in the supplemental material)*.*

10.1128/mSphere.00013-15.3 Figure S1  Mutant strains exhibit wild-type level of growth in laboratory medium. Growth kinetics of parental and genetically defined mutant strains in lysogeny broth are reported here as increase in absorbance over time from three independent experiments. Absorbance of the culture was measured at 30-min intervals in the Bioscreen C system, as described in Materials and Methods of the article. Error bars indicate standard errors of the means (SEM). Download Figure S1, TIF file, 0.2 MB. Copyright © 2015 Subashchandrabose et al. 2015 Subashchandrabose et al. This content is distributed under the terms of the Creative Commons Attribution 4.0 International license.

The wild-type strain was mixed 1:1 with each of the *feoB*, *pntB*, and *ddc* mutant strains and used to inoculate leukopenic mice intravenously. Mice were euthanized at 24 hpi, spleens and livers were collected, and homogenates were plated to determine the numbers of wild-type and mutant bacteria. Competitive indices were calculated as the ratio of mutant over wild type in the spleen at 24 hpi to the ratio of mutant over wild type in the inoculum. Competitive indices of <1 indicate that a given mutant exhibits a fitness defect relative to the fitness of the parental strain (Fig. 2B). Mutants lacking *feoB* and *pntB* were outcompeted by the wild-type strain in both spleen and liver (Fig. 2B). The *ddc* mutant exhibited a fitness defect in the liver but not in the spleen (Fig. 2B). These coinfection experiments demonstrate that *feoB*, *pntB*, and *ddc* genes are involved in the fitness of *A. baumannii* during systemic colonization resulting from bacteremia. To elucidate the possible mechanisms by which these genes contribute to fitness, *in vitro* assays were performed using genetically defined mutants to assess iron acquisition potential, survival in serum, resistance to antimicrobial peptides, and intracellular survival in macrophages.

### FepA is involved in enterobactin uptake.

**FIG 3:**
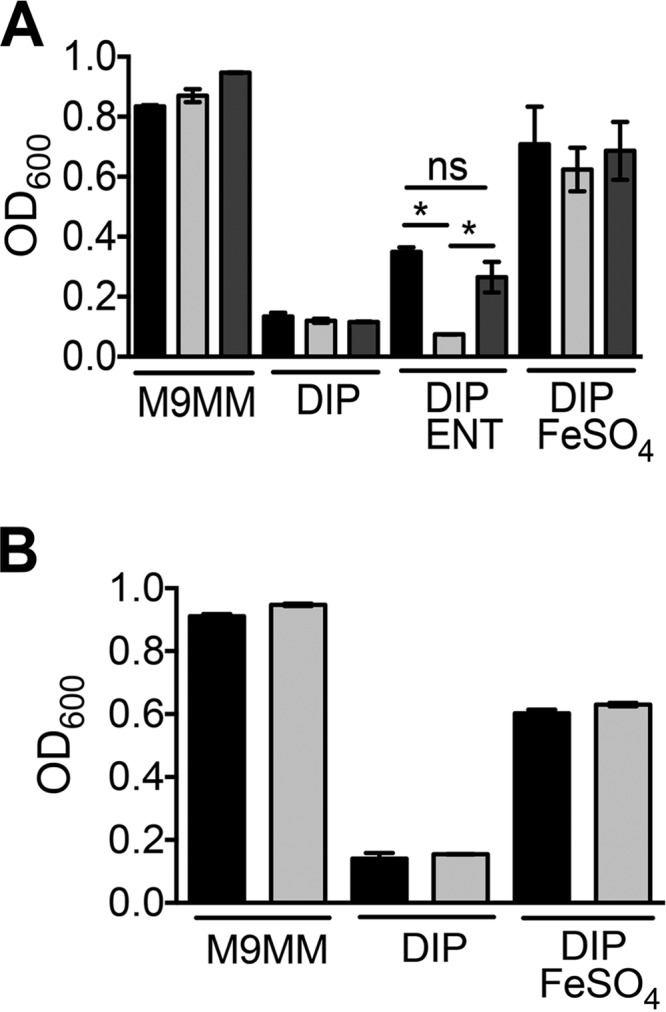
FepA is required for enterobactin uptake but FeoB is dispensable for ferrous iron uptake. Wild-type and mutant strains were grown in M9 minimal medium (M9MM) for 22 h in the presence of 200 µM dipyridyl (DIP) and 1 µM enterobactin (ENT) or 1 µM FeSO_4_. (A) The wild-type (black bars), *fepA* mutant (light gray bars), and complemented mutant (dark gray bars) strains reached comparable optical densities in stationary phase in M9MM, M9MM plus DIP, and M9MM plus DIP and FeSO_4_. The *fepA* mutant exhibited a growth defect only in medium supplemented with enterobactin, whereas the wild-type (WT) and complemented mutant strains were capable of utilizing exogenous enterobactin, as reflected by higher levels of growth. (B) The wild-type (black bars) and *feoB* mutant (light gray bars) strains reached comparable optical densities in stationary phase in M9MM, M9MM plus DIP, and M9MM plus DIP and FeSO_4_. The experiments were repeated three times independently. Bars indicate mean values, and error bars indicate standard errors of the means (SEM). Statistical significance was tested using one-way analysis of variance (ANOVA), followed by Sidak’s test for multiple comparisons (*, <0.05). OD_600_, optical density at 600 nm.

Two genes, *fepA* and *feoB*, proposed to be involved in iron uptake based on sequence homology-based annotation, were identified as augmenting fitness during bacteremia (see Table S1 in the supplemental material). Although *A. baumannii* ATCC 17978 encodes a receptor for ferric enterobactin, this strain lacks the genes encoding enterobactin biosynthetic machinery. Wild-type, *fepA*, and *feoB* mutant strains exhibited comparable growth in minimal medium or in minimal medium containing 100 µM dipyridyl, an iron chelator (Fig. 3A and B). The addition of 1 µM enterobactin, a catecholate siderophore, to iron-chelated medium rescued the growth of the wild type but not the *fepA* mutant, indicating that *fepA* encodes a receptor that is involved in importing enterobactin (Fig. 3A). Genetic complementation with *fepA* and *A1S_0981* genes, including the native promoter, rescued the growth defect of *fepA* mutant in the presence of exogenous enterobactin, confirming that FepA is required for successful utilization of enterobactin as an iron source during culture in iron-chelated medium (Fig. 3A). Upon iron (1 µM FeSO_4_) supplementation in iron-chelated medium, the *fepA* mutant reached the wild-type level of growth, indicating that the mutant does not have an inherent growth defect during culture in iron-chelated medium (Fig. 3A).

The wild-type strain and the *feoB* mutant exhibited comparable growth rates in minimal medium, iron-chelated minimal medium, and iron-chelated medium supplemented (1 µM FeSO_4_) with iron. Taken together, these results suggest the presence of additional ferrous iron importers in *A. baumannii* (Fig. 3B)*.*

### Survival in human serum.

**FIG 4:**
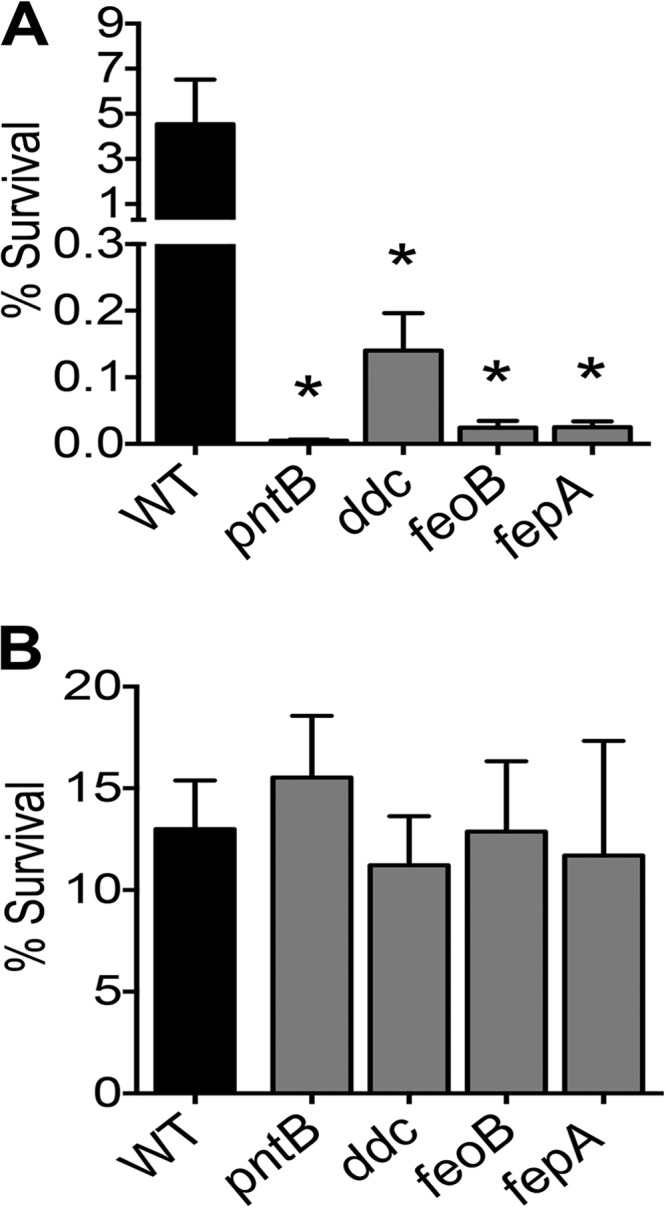
Bacteremia fitness genes augment resistance against human complement. Survival of wild-type and mutant strains in 90% human serum (A) and heat-inactivated serum (B) for 30 min. The mutants lacking *pntB*, *ddc*, *feoB*, and *fepA* exhibit decreased survival compared to that of the wild type in serum. However, heat inactivation of serum relieves the survival defect of these mutants. Bars indicate mean rates of survival relative to the sizes of the inocula and error bars indicate SEM from three independent experiments. Statistical significance was tested using one-way ANOVA, followed by Dunnett’s test for multiple comparisons (*, <0.05).

*A. baumannii* fitness genes were identified and validated in a mouse model of bacteremia. Therefore, survival in human serum was used to probe the role of these genes under conditions emulating human bacteremia. Pooled human serum obtained from clinically healthy volunteers for another study (14) was used in this assay. The survival of wild-type and mutant strains in 90% serum and heat-inactivated serum was tested. Heat inactivation is an established method to deplete complement activity, the major bactericidal factor in serum. The wild-type strain exhibited a pronounced loss of viability in serum and a subtle but consistent loss of viability in heat-inactivated serum after 30, 60, and 120 min of exposure (see Fig. S2A in the supplemental material). These results indicate that complement is a major but not the exclusive bactericidal factor against *A. baumannii* during exposure to human serum. The *pntB*, *ddc*, *feoB*, and *fepA* mutants were hypersensitive to serum at 30 and 60 min compared to the sensitivity of the parental strain (Fig. 4A; see also Fig. S2B). However, these mutants exhibited wild-type levels of survival in heat-inactivated serum (Fig. 4B; see also Fig. S2C), indicating that these mutants are highly susceptible to complement-mediated bactericidal activity of human serum. The serum sensitivity phenotype could be rescued, at least partially, by genetic complementation, indicating that these gene products make specific contributions to complement resistance (see Fig. S2D).

10.1128/mSphere.00013-15.4 Figure S2  Serum survival assays and genetic complementation to rescue serum survival. (A) Survival of *A. baumannii* strain ATCC 17978 in 90% serum and heat-inactivated serum at 37°C was determined in pooled human sera. Heat-inactivated serum was used to delineate complement-independent effect of *A. baumannii* survival in sera. Survival of WT and mutant strains in 90% serum (B) and heat-inactivated serum (C) for 60 min at 37°C is presented here. Heat inactivation rescues the survival defect of bacteremia fitness gene mutants. (D) Genetic complementation promoted survival in 50% serum for *pntB* mutant. For other mutants, complementation does not fully rescue the growth defect phenotype. Mean values and SEM presented here are from at least three independent experiments. Statistical significance was tested using one-way ANOVA, followed by Dunnett’s test for multiple comparisons (*, <0.05). Download Figure S2, TIF file, 0.6 MB. Copyright © 2015 Subashchandrabose et al. 2015 Subashchandrabose et al. This content is distributed under the terms of the Creative Commons Attribution 4.0 International license.

### Resistance to antimicrobial peptides.

**FIG 5:**
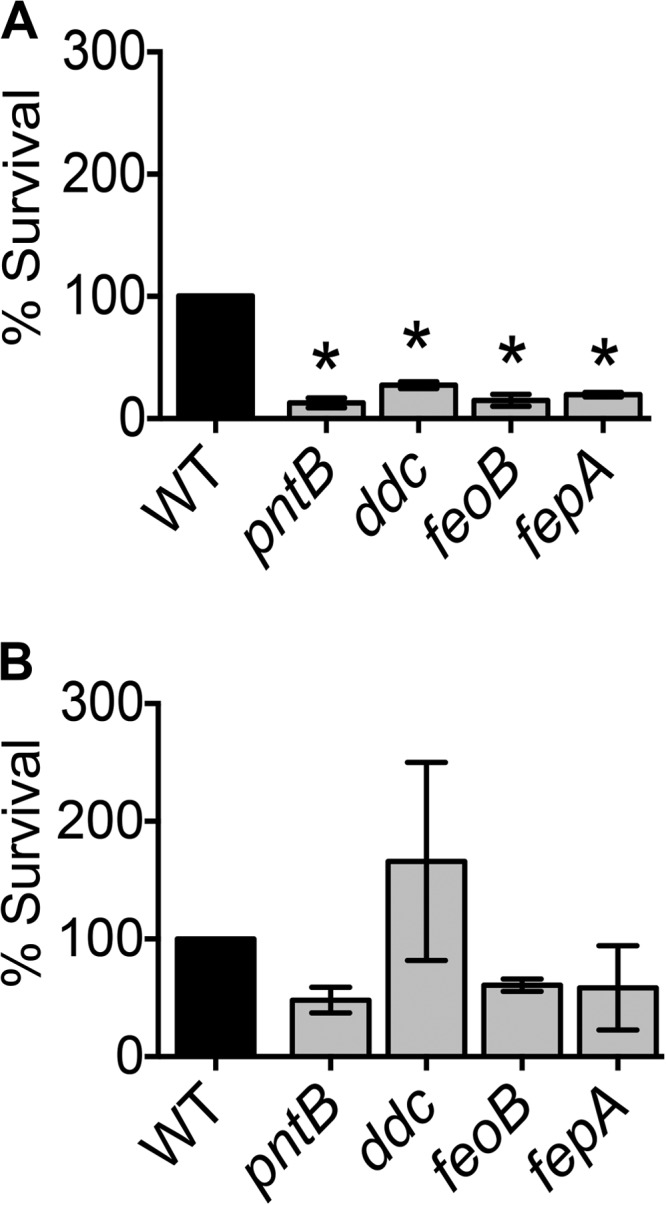
Polymyxin B sensitivity assay. Polymyxin B was used as a surrogate for assessing resistance to host-derived antimicrobial peptides. (A) Compared to the wild-type strain, *pntB*, *ddc*, *feoB*, and *fepA* mutants are hypersensitive to killing by polymyxin B. (B) Genetic complementation rescues resistance to polymyxin B, resulting in survival rates similar to that of the wild type. Mean values and SEM from at least three independent experiments are presented here. *P* values were determined by one-way ANOVA, followed by Dunnett’s test for multiple comparisons (*, <0.05).

Antimicrobial peptides are an integral component of innate immune response to bacterial infection. Polymyxin B is a peptide antibiotic that mimics the activity of host-derived cationic antimicrobial peptides and was used as a surrogate to test resistance to antimicrobial peptides. The *pntB*, *ddc*, *feoB*, and *fepA* mutants were hypersensitive to polymyxin B compared to the sensitivity of the parental strain (Fig. 5A). Genetic complementation rescued polymyxin B resistance (Fig. 5B), indicating that these gene products are specifically involved in mitigating polymyxin B toxicity in *A. baumannii.*

### Survival in mouse macrophages.

**FIG 6:**
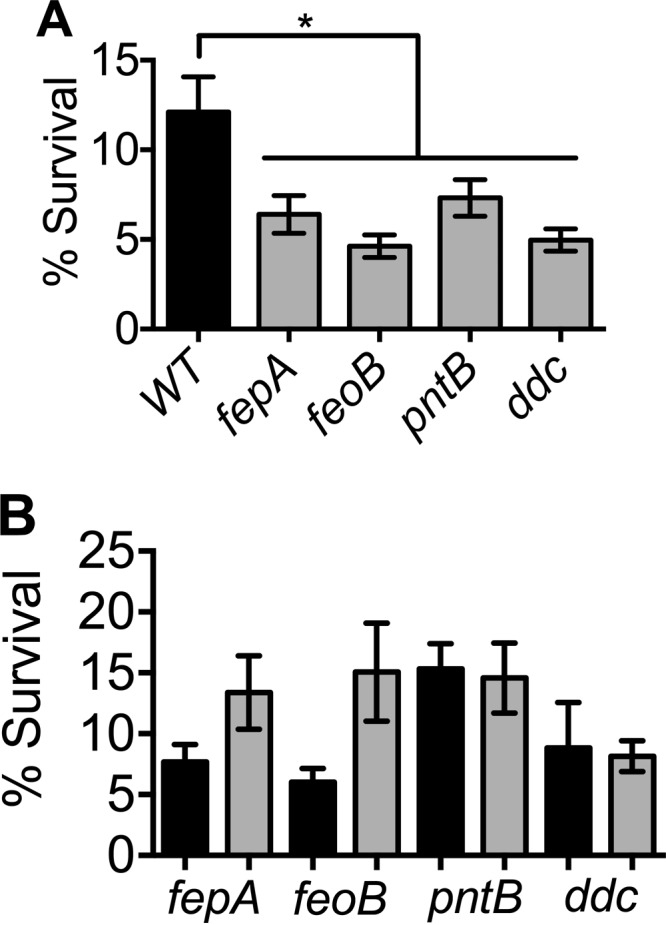
Bacteremia fitness genes are involved in intramacrophage survival. (A) Loss of genes required for bacteremia and subsequent systemic colonization results in survival defects compared to the survival of the parental wild-type strain within murine macrophage cell line RAW 264.7. (B) Complementation reveals a trend toward enhanced survival for *fepA* and *feoB* mutants but not for *pntB* and *ddc* mutants within RAW cells. Results for mutant and complemented mutant strains are shown by black and gray bars, respectively. Mean values and SEM from three independent experiments were analyzed by one-way ANOVA, followed by Dunnett’s test for multiple comparisons (*, <0.05).

Macrophages are key professional phagocytes involved in clearing bacteria during infection. To understand the role of the fitness genes identified in this study, the survival of wild-type and mutant strains of *A. baumannii* in mouse macrophages was measured using immortalized murine macrophage RAW 264.7 cells. The gentamicin protection assay was used to test whether *A. baumannii* mutants with impaired *in vivo* fitness are compromised in intramacrophage survival. The *pntB*, *ddc*, *feoB*, and *fepA* mutants were defective in intramacrophage survival, as evidenced by increased killing, compared to the survival of the parental strain (Fig. 6A). Genetic complementation did not completely rescue the intracellular growth defect (Fig. 6B). Complementation with *fepA* and *feoB* revealed a trend toward enhanced survival, whereas complementation with *pntB* and *ddc* did not appear to affect intracellular survival. These results indicate that the bacteremia fitness genes identified in this study contribute to survival within murine macrophages.

## DISCUSSION

*A. baumannii* is emerging as an opportunistic pathogen that poses a significant public health risk globally (3). In immunocompromised, traumatized, or diabetic patients or those suffering from chronic lung disease, the high prevalence of infection is disturbing because multiple antibiotic resistance of the organism often leaves few or, in some cases, no antimicrobial treatment options (1, 15). Therefore, the situation clearly warrants detailed studies to unravel the mechanisms of pathogenesis of *A. baumannii* and identify virulence genes that could be targeted to selectively disarm this pathogen without complete killing.

Targeted studies have identified a few virulence factors in *A. baumannii*, including an outer membrane protein A (OmpA) (16, 17), phospholipase D (18), the surface polysaccharide poly-*N*-acetylglucosamine (19), a trimeric autotransporter protein (Ata) (20), penicillin-binding proteins 7 and 8 (21), EpsA and a protein tyrosine kinase protein involved in the biogenesis of K1 capsule (22), a zinc acquisition system (23), and iron acquisition systems (24, 25). Additionally, genes involved in iron acquisition, type IV pili biogenesis, competence, and efflux pumps are upregulated in the serum-resistant *A. baumannii* strain 98-37-09 during growth in human serum (26). The role of these genes in resistance to killing in serum, however, remains to be tested. *A. baumannii* genes essential for survival in human ascites fluid have been described recently (27). A total of 18 principally metabolic genes that contribute to growth in ascites fluid were identified as essential for survival in a rat model of subcutaneous abscess (27).

High-throughput sequencing enabled mapping of transposon mutant insertion sites has emerged as a powerful tool to study bacterial gene function at a population level in any niche. Here we use an adaptation of transposon directed insertion-site sequencing that was originally developed in *Salmonella enterica* serovar Typhi (28) to detect fitness of *A. baumannii* genes involved in survival during systemic colonization following bacteremia in a mouse model. Recently, a similar approach was used to detect fitness genes in *A. baumannii* in a mouse model of pneumonia (29). Seven genes (*purL*, *guaA*, *A1S_0419*, *A1S_2315*, *A1S_2585*, *A1S_2768*, and *A1S_3455*) identified in the current study were also reported by Wang et al. (29) to contribute to persistence in the lungs during pneumonia, suggesting the presence of a core set of fitness genes involved in fitness during survival in the murine host, irrespective of the site of infection.

In this study, stringent criteria were applied to detect fitness genes. While the original transposon mutant pool was comprised of 109,000 transformants, the mutant pools were passaged two times in laboratory medium to enrich for mutants lacking a growth defect *in vitro*, followed by three passages through the mouse model of bacteremia. Utilizing this rigorous screen, we were able to identify 2,614 unique insertion sites. It is likely that these steps have reduced the complexity of the transposon mutant pool. However, all mutants detected in the inoculum were also detected in the output from the third passage in mice, albeit at lower levels than in the inoculum, indicating that there is no significant bottleneck associated with the mouse model of bacteremia. Future work utilizing a more complex transposon mutant library and comparing the fitness of mutants derived from multiple passages in mice would be of interest for comparison to the current study.

Since two genes involved in iron uptake (*fepA* and *feoB*) were identified in our screen, we further investigated their role in *A. baumannii* pathogenesis. Similar to observations in many bacterial pathogens, *A. baumannii* encodes multiple diverse iron acquisition systems (25). Therefore, our finding that *fepA* and *feoB* mutants exhibit wild-type levels of growth in iron-chelated medium is not surprising. Indeed, previous work revealed that multiple iron uptake systems are upregulated in response to iron limitation in *A. baumannii* (30). During cloning to complement *fepA*, an annotation error was noted in strain 17978. The current annotation indicates both *A1S_0980* and *A1S_0981* as ferric enterobactin receptor precursors. Compared to other sequenced *A. baumannii* strains, it appears that both *A1S_0980* and *A1S_0981* in strain 17978 are part of one gene involved in uptake of ferric enterobactin.

Here, we report the identification and characterization of genes involved in iron acquisition (*fepA* and *feoB*), peptidoglycan biogenesis (*ddc*), and proton-translocating transhydrogenase (*pntB*) in fitness *in vivo* and in select *in vitro* assays that emulate conditions encountered during infection of a mammalian host. These four genes, apparently involved in three different functions independently, contribute to survival in various milieus. It would be of interest to investigate how these gene functions interact at the cellular level to orchestrate the survival of *A. baumannii* in a hostile niche. Further investigations of the fitness genes identified in this study should delineate additional novel fitness factors in this major human pathogen and lay the groundwork for characterizing these fitness genes as potential antibacterial targets by developing small-molecule inhibitors.

## MATERIALS AND METHODS

### Bacterial strains, generation of mutants, and culture conditions.

*A. baumannii* strain ATCC 17978 and isogenic mutant derivatives were cultured in lysogeny broth (LB; 1% tryptone, 0.5% yeast extract, and 0.05% NaCl) at 37°C. Kanamycin (12.5 and 25 µg/ml) and carbenicillin (75 µg/ml) were used for selection. Recombineering (13) and homologous recombination (12) were used to generate the mutants used in this study (see Table S3 in the supplemental material). Mutations in *fepA* and *pntB* were introduced by recombineering using the kanamycin resistance cassette from pKD4 (31), as described by Tucker et al. (13). For construction of *fepA* and *ddc* mutants, ~500 bp of the 5′ flanking region, the kanamycin resistance cassette, and ~500 bp of the 3′ flanking region (mutation construct) were cloned in tandem into pUC19. The kanamycin cassette used for cloning into pUC19 was amplified from an *E. coli* EZ Tn*5* transformant. To introduce the *fepA* mutation, the mutation construct was electroporated into ATCC 17978 electrocompetent cells expressing recombineering proteins from pAT02 (13). To generate the *ddc* mutant, the mutation construct was electroporated into ATCC 17978 electrocompetent cells as described by Aranda et al. (12). The genetic architecture at the mutated locus was verified by PCR using primers listed in Table S4 in the supplemental material. The growth kinetics of wild-type and mutant strains in LB were determined by measuring the optical density at 600 nM. The Bioscreen C system was used to record the optical densities of 300-µl cultures at 30-min intervals for 14 h. Plates were incubated at 37°C with continuous shaking, and the experiment was repeated three times, independently. Gene sequences comprising ~500 bp of the 5′ flanking region, including the native promoter (*pntB* and *ddc*), *A1S_0980* and *A1S_0981* genes (*fepA*), and the *feoB* and *feoA* genes (*feoA*) were cloned into pABBR_MCS for complementation (13). The oligonucleotide primers used for generation and verification of mutants and complementation plasmids are listed in Table S4.

10.1128/mSphere.00013-15.5 Table S3  Bacterial strains and plasmids used in this study. Download Table S3, PDF file, 0.01 MB. Copyright © 2015 Subashchandrabose et al. 2015 Subashchandrabose et al. This content is distributed under the terms of the Creative Commons Attribution 4.0 International license.

10.1128/mSphere.00013-15.6 Table S4  Oligonucleotide primers used in this study. Download Table S4, XLSX file, 0.01 MB. Copyright © 2015 Subashchandrabose et al. 2015 Subashchandrabose et al. This content is distributed under the terms of the Creative Commons Attribution 4.0 International license.

### Generation of high-density transposon mutant library.

Random Tn*5* transposon insertion mutants were generated in *A. baumannii* strain ATCC 17978, a sequenced clinical strain isolated from a neonate suffering from meningitis (32). Based on the size of the genome, 45,995 transposon insertion mutants are required to achieve 99.99% genome saturation coverage (33). Tn*5*-transposase complexes (R6Kγori/KAN-2 Tnp transposome kit; Epicentre) were electroporated into *A. baumannii* strain ATCC 17978 electrocompetent cells, and kanamycin-resistant transformants were selected. PCR with transposon-specific primers was used to verify that kanamycin-resistant transformants indeed contained the transposon and there was no amplification from the wild-type strain (see Table S4 in the supplemental material). Considering the possibility of redundant insertions and insertional hot spots, a total of 109,000 mutants were generated, which provides >2 times the number of mutants required for 99.99% genome coverage. The mutant pool was passaged twice in LB at 37°C to enrich for mutants that did not exhibit severe growth defects *in vitro*, and this enriched mutant pool was archived and used for infection experiments.

### A leukopenic mouse model.

All procedures involving the use of mice were approved by the University Committee on Use and Care of Animals at the University of Michigan. A low-dose cyclophosphamide (CP) treatment regimen (11) was used to induce leukopenia in 5- to 6-week-old CBA/J mice (Harlan Laboratories). Briefly, mice were injected intraperitoneally with CP doses of 150 mg and 100 mg/kg of body weight at 4 days and 3 days prior to infection, respectively. Control mice received phosphate-buffered saline (PBS). Blood samples were collected before and two days after treatment with CP or PBS. Hematology profiles were determined using a Hemavet 950FS system at the Unit for Laboratory Animal Medicine at the University of Michigan. Mice were inoculated with a 100-µl inoculum containing 10^7^ CFU of the wild-type strain or transposon mutants via tail vein. Spleens and livers were collected and homogenized in 3 ml PBS. Homogenates were plated on LB with kanamycin to determine colonization levels (Fig. 2). The same procedure was repeated using the transposon mutant pool and bacteria derived from the spleen homogenates as the inocula for the next round of infection. A total of three rounds of infection were performed to select against mutants that exhibit a fitness or virulence defect within murine hosts. The inoculum used for the first set of infections (input) and bacteria cultured from mouse spleens isolated in the third set of infections (output) were used for genomic DNA isolation.

### Illumina sequencing.

Genomic DNA was extracted from the input and output pools using the DNeasy genomic DNA isolation kit (Qiagen). DNA (5 µg) was used for Illumina TruSeq library preparation according to manufacturer’s instructions. Briefly, genomic DNA was sheared and ~250-bp fragments were size selected on a gel and used for adapter ligation. Libraries were amplified by PCR with a Tn-specific forward primer and a custom Illumina reverse primer to amplify transposon-genomic DNA junctions (see Table S4 in the supplemental material). Transposon-genomic DNA junctions were sequenced with a Tn-specific sequencing primer (see Table S4) in an Illumina HiSeq 2000 sequencer at the University of Michigan DNA core facility, using a 50-nucleotide single-end read cycle.

### Determination of genes involved in survival during bacteremia.

Genomic DNA isolated from the inoculum and from bacteria derived from spleens (third passage) was used to generate sequencing libraries. The libraries were enriched for Tn-genomic DNA junctions by PCR and sequenced with a Tn-specific primer. Illumina reads from the input (30,279,583) and output (57,590,410) libraries were mapped to the genome of *A. baumannii* strain ATCC 17978 (NCBI genome accession number CP000521) using the short-read aligner BOWTIE (34). The frequency of detection of Tn insertion at all insertion sites was determined using TFAST (35), as previously described for a uropathogenic *E. coli* transposon library (10). Reads were normalized to the total reads obtained from the sample, and a fitness factor was calculated for each insertion site as the ratio of the normalized frequency of a Tn insertion mutant in the input to that of the output.

### Coinfection experiments.

Inocula were prepared as described above with one modification: equal volumes of wild-type and mutant strain suspensions were mixed prior to inoculation. Mice were inoculated with a 100-µl inoculum containing 10^7^ CFU of the wild type and a mutant strain via the tail vein. Mice were rendered leukopenic as described above, and 10 mice were used in each experiment. Spleens and livers were collected at 24 hpi. Homogenates were plated on LB and LB with kanamycin to enumerate CFU of the wild type and the mutants. Competitive indices were calculated as the ratio of mutant bacteria over the wild type in tissue divided by the ratio of mutant bacteria over the wild type in the input.

### Culture in iron-limiting medium.

Strains were cultured overnight in LB and passaged twice in M9 minimal medium (M9MM: 50 mM Na_2_HPO_4_ 7H_2_O, 20 mM KH_2_PO_4_, 20-mM glucose, 20 mM NH_4_Cl, 8.5 mM NaCl, 2 mM MgSO_4_, 100 µM CaCl_2_, and 0.2% Casamino acids) for 24 h each time prior to each experiment. Strains carrying complementation plasmids or the empty vector (pABBR_MCS) were always cultured with carbenicillin. Dipyridyl, enterobactin, and FeSO_4_ were used at final concentrations of 100 µM, 1 µM, and 1 µM, respectively. The optical densities of 300-µl cultures were measured at 600 nm in a Bioscreen C system. The experiment was repeated three times independently.

### Serum survival assay.

Survival of the wild type and mutants of *A. baumannii* in pooled human serum was determined at 37°C. Overnight cultures were diluted 1:1,000 in PBS and diluted 1:10 to a final volume of 100 µl in 90% serum or 90% heat-inactivated serum. Serum was heat inactivated by incubating at 56°C for 30 min. Dilutions were plated at 0, 30, 60, 90, 120, and 240 min. Samples were analyzed in triplicate, and the entire experiment was repeated three times independently. Strains carrying complementation plasmids or empty vector (pABBR_MCS) were cultured overnight in LB with carbenicillin, but antibiotic selection was not used during the serum survival assay. For complementation, survival was determined in 50% serum due to the extremely poor survival of mutant strains in 90% serum after 30 min of exposure. Percent survival was calculated as the fraction of the original inoculum that survived at multiple endpoints tested here.

### Polymyxin B survival assay.

The survival of the wild type and mutants of *A. baumannii* in LB medium containing polymyxin B (2 µg/ml) was determined at 37°C. Overnight cultures were diluted 1:100 into fresh LB medium and incubated for 1 h prior to the addition of polymyxin B. Dilutions were plated at 0 and 10 min posttreatment. Samples were analyzed in duplicate, and the entire experiment was repeated three times independently. Strains carrying complementation plasmids or empty vector (pABBR_MCS) were cultured overnight in LB medium with carbenicillin, but antibiotic selection was not used during the polymyxin B survival assay. Percent survival was calculated as the fraction of the original inoculum that survived at the 10-min end point, and the percent survival for the wild type was set at 100%.

### Intramacrophage survival assay.

Mouse macrophage cell line RAW 264.7 was cultured in RPMI 1640 supplemented with 1× penicillin, streptomycin, and l-glutamine (Invitrogen) and 5% fetal bovine serum (Sigma) and seeded in 48-well plates 24 h prior to the experiment. Overnight cultures were diluted 1:10 in PBS and further diluted 1:10 in RPMI 1640. RAW cells were washed twice in PBS prior to the addition of inocula. Two identical plates were set up for each experiment for *T*_0_ and *T*_2_ time points (described below); each strain was tested in triplicate, and the entire experiment was repeated three times. The plates were incubated for 30 min to allow phagocytosis, followed by removal of supernatants and the addition of RPMI 1640 with gentamicin (200 µg/ml). *T*_0_ samples were collected after 10 min of gentamicin treatment that killed extracellular bacteria. *T*_2_ samples were collected 2 h after the addition of gentamicin. At both time points, supernatant was aspirated prior to washing monolayers with PBS. RAW cells were lysed with 0.1% saponin in autoclaved water and plated to enumerate bacteria within the macrophages. Percent survival was calculated as the fraction of the original intramacrophage bacterial population (*T*_0_) that survived for an additional 2 h within RAW cells (*T*_2_).

### Data accession number

The raw reads can be accessed in the NCBI Sequence Read Archive under the SRA accession number SRP062998 .
