# Supplementary figures and images for: Acinetobacter baumannii Genes Required for Bacterial Survival during Bloodstream Infection
Source: mSphere. 2015 Nov 4;1(1):e00013-15. doi: 10.1128/mSphere.00013-15 (PMC4863628; doi:10.1128/mSphere.00013-15)

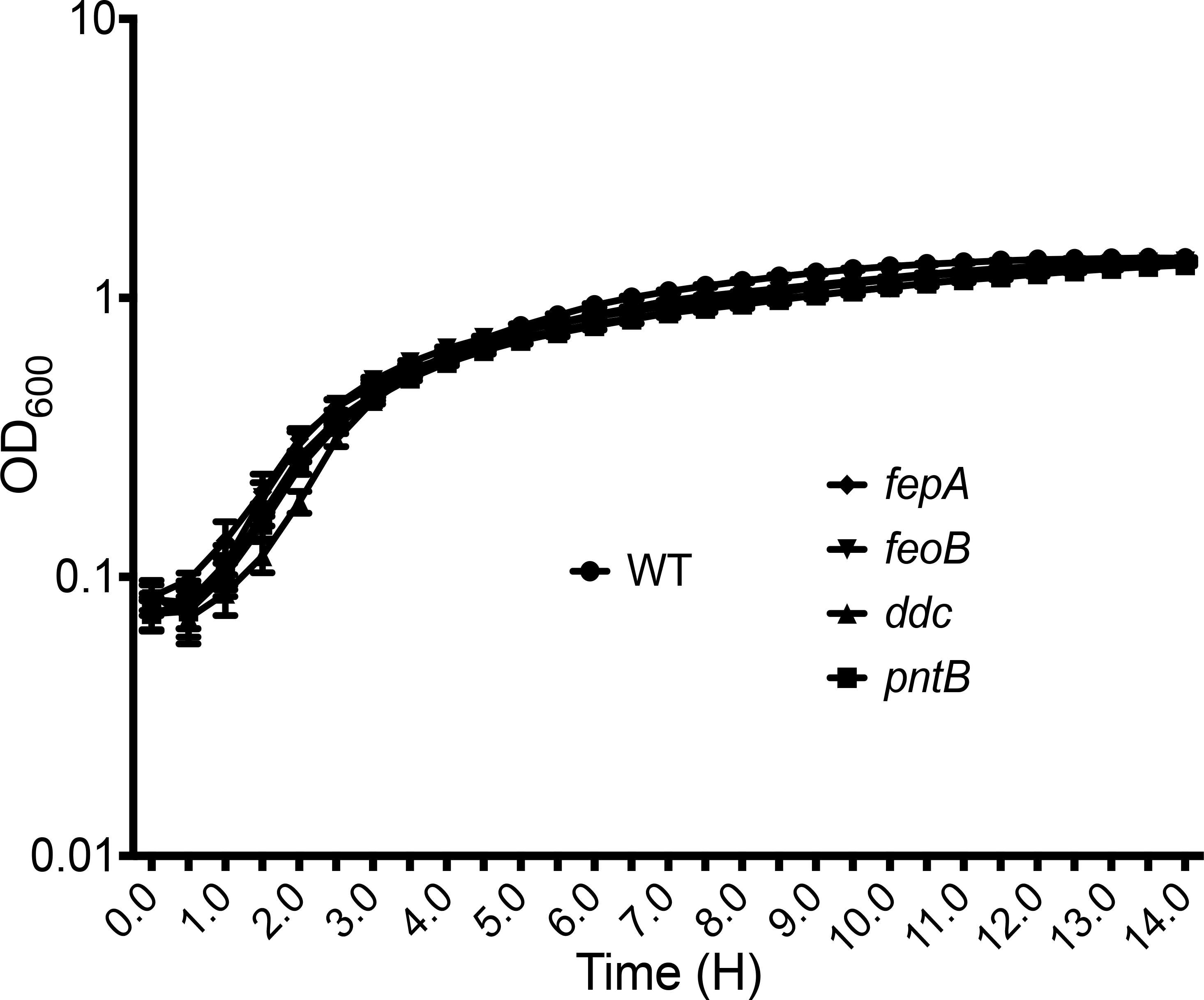

Supplement: Figure S1 [file sph001160011sf1.tif]

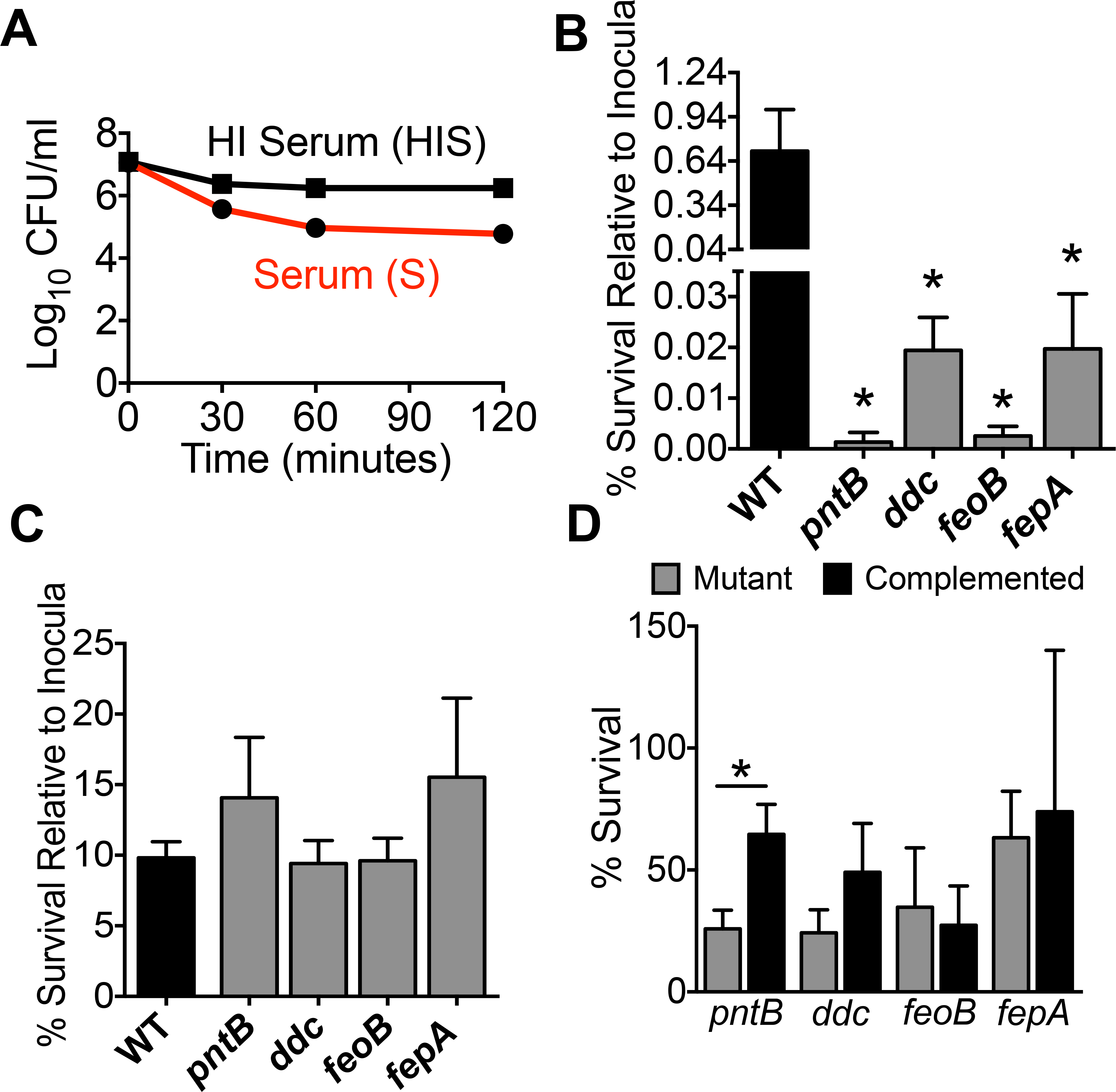

Supplement: Figure S2 [file sph001160011sf2.tif]
