# Supplementary material for: Acinetobacter baumannii Genes Required for Bacterial Survival during Bloodstream Infection
Source: mSphere. 2015 Nov 4;1(1):e00013-15. doi: 10.1128/mSphere.00013-15 (PMC4863628; doi:10.1128/mSphere.00013-15)
Supplement: Table S3 [file sph001160011st5.pdf]

Table S3. Bacterial strains and plasmids used in this study

Strain/Plasmid	Genotype/Resistance/Use[a]	Source
**Strains**		
A. baumannii strain 17978	wild-type	ATCC
fepA (*A1S_0980*)	17978 Δ*fepA::*kan^{R}	This study
fepA+	*fepA* with PABBR_MCS_*fepA_A1S_0981*	This study
feoB (*A1S_0653*)	17978 Δ*feoB::*kan^{R}	This study
feoB+	*feoB* with PABBR_MCS_ *A1S_0652_feoB*	This study
ddc (*A1S_0568*)	17978 Δ*ddc::*kan^{R}	This study
ddc+	*ddc* with PABBR_MCS_*ddc*	This study
pntB (*A1S_02435*)	17978 Δ*pntB::*kan^{R}	This study
pntB+	*pntB* with PABBR_MCS_*pntB*	This study
Escherichia coli EZTn*5*	Source of kanamycin cassette	S. Subashchandrabose
**Plasmids**		
pUC19	amp^{R}, cloning mutant construct	Life Technologies
pAT02	Rec$_{Ab}$ system, recombineering	13
pKD4	kan^{R}, source of kanamycin cassette	31
PABBR_MCS	amp^{R}, cloning for complementation	13
PABBR_MCS_*fepA_A1S_0981*	amp^{R}, complementation	This study
PABBR_MCS_*feoB_ A1S_0652*	amp^{R}, complementation	This study
PABBR_MCS_*ddc*	amp^{R}, complementation	This study
PABBR_MCS_*pntB*	amp^{R}, complementation	This study

[a]kan, kanamycin; amp, ampicillin/carbenicillin, R, resistant
